# Siamese Connection Successfully Dissevered

**Published:** 1835

**Authors:** Delos White

**Affiliations:** Professor of Anatomy in the Western Medical College of New York


### III.—Siamese Connection Successfully Dissevered.

*A case of twins united at the umbilicus, in which the connection was cut and the perfect child survived.* By Delos White, M. D., Professor of Anatomy in the Western Medical College of New York. Communicated by George W. Little, M. D.

*To the Editor of the Western Medical and Surgical Journal:*

Sir—Believing that the particulars of the following unpublished case may possess some interest for your readers, I take the liberty of sending you a sketch of it. It goes, I think, fully to establish one important fact, which, as far as I know, has been considered somewhat questionable by operators—viz: the capability of very young infants to sustain formidable operations. It is believed, also, that the case will be found interesting in other points of view.

Mrs. W———, of Otsego county, N. York, the mother of several healthy well formed children, was taken in labor in the month of November, 1833. The presentation was natural, and the labor proceeded without any unusual difficulty, until the head and shoulders were expelled. There was then delay in the further delivering, owing to some cause which the attending physician could not readily understand. After a few severe pains, however, the labor was completed, and the cause of the delay became sufficiently obvious. The child presented to the astonished eyes of the accoucheur the appearance of being *double below the sternum.*

The parents were naturally exceedingly distressed at the deformity, and very anxious that if possible something should be done to remove it. The father, soon after the birth of the child, called upon Dr. Delos White, of Cherry Valley, with whom I was then a partner in business, stating as nearly as he could the particulars of the ease, and requested the Dr. to examine it and give his opinion whether the redundant parts could be removed without imminently endangering the life of the child. In compliance with that request we made a particular examination of the case.

We found attached to the scrobiculis cordis a perfectly formed pelvis and lower limbs of a male child, with the parts of generation perfectly developed. The connecting medium between the parasitical parts and the child was about two inches long, two and a half inches wide, and one and a half inch thick. A little higher than the centre of this medium of communication, and deep in its substance, a hard substance could be felt, extending from the body of the child about an inch and a half, and seemed to be formed by the prolongation of the ensiform cartilage. The limbs had no muscular motion, but lay bent upon themselves and crossed upon each other. The sense of feeling, however, existed in them as perfectly as in the other limbs. There was no trace of the anus. Urine was discharged once in two or three days in small quantities. The parasite was plump and perspirable and exhibited every indication of being amply nourished. It was moveable, and could be turned one quarter round without difficulty, and without producing any inconvenience to the child—when the rotation was attempted to be carried farther the child cried from pain. The redundant parts were found upon measurement to be as large in all their dimensions as the corresponding parts of the child. Upon a careful examination of the connecting medium we could distinctly feel the pulsation of an artery apparently of large size. When the examination was concluded, Dr. White gave as his opinion to the parents, that a separation by the knife was the only mode of remedying the evil, and that the operation, though unquestionably hazardous, was not, in his judgment, necessarily fatal—he left the decision of the question whether it should be done, entirely to themselves, but remarked, that if it was a child of his own he should perform the operation. After a little consultation the parents consented to the operation, and the time was fixed for the performance of it four weeks from that time, when the child would be six weeks old.

On the day fixed the separation was made. The child was remarkably vigorous and healthy, and it was the opinion of the mother that the parasitical parts were growing faster than the others. The patient was undressed and held in the lap of an assistant, and two elliptical incisions meeting at their extremities were made on the connecting medium about three-fourths of an inch from the body of the child. The skin and subjacent tissue were found exceedingly vascular, and considerable blood was lost from minute vessels—the hard substance mentioned above was found to be as was supposed, cartilaginous, of an exceedingly fine texture, approaching to ossification. It was of course cut through during the operation. About the centre of the connection, immediately below the cartilage above mentioned, was observed a kind of sheath, which, upon being cautiously opened, was found to contain an artery, vein, and nerve of considerable size. The artery and vein were tied, separately and divided, and the separation was completed. Several vessels were tied during the operation, which, though small, bled very smartly. The child was very faint, and much exhausted after the operation, (which occupied more than a quarter of an hour,) and it was with the greatest difficulty the vital spark could be maintained by the exhibition of cordials, stimulants, etc. After the lapse of a few hours it revived, and passed the night very comfortably. No dissection was made of the parts removed—they weighed two pounds six ounces. The substance divided by the knife consisted of the common integument—a thin layer of very vascular cellular membrane—the cartilage mentioned above—and a portion of fleshy substance in which muscular fibres could be very indistinctly traced. On the third day the dressings were removed, adhesion had already taken place to some extent, and the wound seemed to be doing well. From this time the little patient rapidly progressed to recovery, without the occurrence of a single bad symptom, and is now as healthy and as free from deformity as any child in existence.

It may be proper to notice, that while in the seventh month of pregnancy, the mother of this child became much interested in the story of the Siamese twins, who were about that time being exhibited in this section of country. She went on one occasion to our county town (when they were expected,) to see them. They were not there, however, and her curiosity was not gratified. The subject continued to occupy her thoughts in spite of her efforts to banish it for a long time. What influence this may have had in producing the malformation of the fœtus with which she was then pregnant, is left to the curious in such matters to decide.

Respectfully yours,

George W. Little.

